# Universal Genotyping for Tuberculosis Prevention Programs: a 5-Year Comparison with On-Request Genotyping

**DOI:** 10.1128/JCM.01778-17

**Published:** 2018-04-25

**Authors:** Jennifer L. Guthrie, Clare Kong, David Roth, Danielle Jorgensen, Mabel Rodrigues, Patrick Tang, Maichael Thejoe, Kevin Elwood, Victoria J. Cook, James Johnston, Jennifer L. Gardy

**Affiliations:** a School of Population and Public Health, University of British Columbia, Vancouver, Canada; b British Columbia Centre for Disease Control Public Health Laboratory, Vancouver, Canada; c British Columbia Centre for Disease Control, Vancouver, Canada; d Department of Pathology and Laboratory Medicine, University of British Columbia, Vancouver, Canada; e Department of Medicine, University of British Columbia, Vancouver, Canada; Carter BloodCare & Baylor University Medical Center

**Keywords:** MIRU-VNTR genotyping, program assessment, tuberculosis

## Abstract

Prospective universal genotyping of tuberculosis (TB) isolates is used by many laboratories to detect clusters of cases and inform contact investigations. Prior to universal genotyping, most TB prevention programs genotyped isolates on request only, relying on requests from public health professionals whose knowledge of a patient's clinical, demographic, and epidemiological characteristics suggested potential transmission. To justify the switch from on-request to universal genotyping—particularly in the public health domain, with its limited resources and competing priorities—it is important to demonstrate the additional benefit provided by a universal genotyping program. We compared the clustering patterns revealed by retrospective 24-locus mycobacterial interspersed repetitive unit–variable-number tandem repeat genotyping of all culture-positive isolates over a 5-year period to the patterns previously established by our genotyping-on-request program in the low-incidence setting of British Columbia, Canada. We found that 23.8% of isolates were requested during the study period, and while requested isolates had increased odds of belonging to a genotype cluster (adjusted odds ratio, 2.3; 95% confidence interval, 1.5 to 3.3), only 54.6% clustered with the requested comparator strain. Universal genotyping revealed 94 clusters ranging in size from 2 to 53 isolates (mean = 5) and involving 432 individuals. On-request genotyping missed 54 (57.4%) of these clusters and 130 (30.1%) clustered individuals. Our results underscore that TB patient networks are complex, with unrecognized linkages between patients, and a prospective province-wide universal genotyping program provides an informative, bias-free tool to explore transmission to a degree not possible with on-request genotyping.

## INTRODUCTION

Despite declining case rates, tuberculosis (TB) remains a public health issue in Canada and other low-incidence countries (1). Here, a substantial proportion of TB diagnoses occur in foreign-born persons and represent reactivation of latent TB infection (1, 2). However, outbreaks and endemically circulating strains also contribute to incidence rates (3–5). Interruption of these transmission chains requires an understanding of regional epidemiology. Techniques such as 24-locus mycobacterial interspersed repetitive unit–variable-number tandem repeat (MIRU-VNTR) genotyping can provide valuable insights into the potential extent of local TB transmission by using clustering as a proxy; thus, many low-incidence settings have incorporated MIRU-VNTR genotyping into standard practice (6–9).

Several laboratories now perform universal genotyping (7, 9–13), in which all culture-positive isolates from a region are prospectively genotyped by one or more molecular methods. While published reports have examined clustering rates and other metrics related to universal genotyping programs (14–16), there are no reports directly comparing the results of universal genotyping to those of an on-request genotyping program over the same time period in the same region.

In the Province of British Columbia (BC), Canada, Mycobacterium tuberculosis isolates are MIRU-VNTR genotyped by the BC Centre for Disease Control (BCCDC) Public Health Laboratory (PHL). From 2009 through 2013, genotyping was done only when requested by BCCDC TB Services personnel. However, a recent province-wide retrospective molecular epidemiology research study later genotyped all culture-positive BC isolates from 2005 to 2014 (*n* = 2,290) to describe the complete genotypic landscape of TB in BC (17). This data set was used to compare the insights derived from the on-request genotyping performed between 2009 and 2013 to those later revealed through genotyping of all of the remaining isolates during this period. Given the significant costs, time, and effort associated with the implementation of universal genotyping, it was important to assess the epidemiological value it adds in a low-incidence setting such as BC, where >75% of TB cases occur in persons born outside Canada and are likely not due to local transmission (17, 18).

## MATERIALS AND METHODS

### On-request genotyping data.

The BCCDC PHL performs routine TB diagnostics, phenotypic drug susceptibility testing, and 24-locus MIRU-VNTR genotyping for all culture-confirmed cases in BC. Until mid-2014, MIRU-VNTR genotyping was performed only when requested by a clinician—typically to support outbreak investigations and contact tracing efforts—with all requests recorded in a spreadsheet. We used this spreadsheet to identify all of the genotyping requests received between 1 January 2009 and 31 December 2013—the last full calendar year before universal genotyping was implemented. On the basis of the information contained in the spreadsheet, we coded the reason for each request as (i) suspected possible transmission, (ii) distinguishing relapse from reinfection, or (iii) suspected false-positive results. For inquiries regarding possible transmission, we noted whether the request asked for comparison to a specific patient(s), to a known outbreak, or to the general database.

### Universal genotyping data.

We have previously described a retrospective genotyping analysis of culture-positive M. tuberculosis isolates diagnosed in BC between 2005 and 2014 (17); here, we examine the subset of isolates received between 2009 and 2013 (*n* = 1,136) and an additional 39 isolates requested for genotyping during this period but from specimens received prior to 2009. For patient-based analyses, the study sample excluded false-positive TB diagnoses (*n* = 3) and the second record of a reoccurrence, leaving a total of 1,158 patients. Briefly, M. tuberculosis sensu stricto isolates were genotyped by standard 24-locus MIRU-VNTR methods (19) and linked to patient level clinical, demographic, and epidemiological data extracted from the BCCDC Integrated Provincial Health Information System (iPHIS) (17). Postal codes were used to obtain the corresponding census dissemination area (DA) for each patient, which we linked to the 2006 Canadian Marginalization Index (CAN-Marg) to determine the deprivation index quintile (20).

### Statistical analysis.

Data were analyzed and presented as means with standard deviations and relative frequencies, as appropriate. We used logistic regression to estimate the odds ratio (OR) and 95% confidence interval (CI) for the association between genotype requested to confirm/refute transmission (yes/no) and MIRU-VNTR genotyping clustered (yes/no). We defined a cluster as ≥2 patients with identical 24-locus MIRU-VNTR genotyping patterns by using a stringent perfect type match, and each cluster was labeled with a unique identifier (MClustID). To obtain the adjusted OR (aOR), we selected variables *a priori*, which included age group, gender, birthplace (Canada/outside Canada), and the presence of one or more risk factors (HIV, drug use, or alcohol misuse) known to be associated with TB transmission and therefore genotype clustering (21). Only patients with complete data for all variables were included in the model (*n* = 910). A secondary analysis was conducted on a subset of the 2009 to 2013 data (2013 quarter 3 [Q3] and Q4 excluded) to explore the possibility that the relationship between genotypic clustering and request status was influenced by the large increase in requests during the last two quarters of 2013. An additional analysis to examine risk factors in relation to genotype requests and clustering status used patient records with complete risk factor data (*n* = 916). Characteristics of all clusters with ≥3 persons (i.e., growing clusters) were analyzed, and the predominant birthplace was assigned as Canada where ≥50% of the persons in the cluster were born in Canada; otherwise, the predominant birthplace was categorized as outside Canada. The cluster growth rate was calculated as the average increase in case counts per quarter over the study period, and linear regression was used to test the relationship of growth rate, cluster size, and birthplace on cluster proportion requested. All analyses were executed in R (v3.3.1).

Ethics approval was granted by the University of BC (certificate H12-00910).

## RESULTS

### The genotype request proportion was smaller than the genotypic clustering proportion.

Our study sample included 1,175 isolates, consisting of 1,136 culture-positive M. tuberculosis specimens received by the BCCDC PHL from 2009 through 2013 and 39 isolates requested during the study period that were received prior to 2009 (see Fig. S1 in the supplemental material). During this time, clinicians submitted 194 genotyping requests involving 309 isolates from 296 patients, including 13 isolates from TB recurrences. The quarterly request proportion varied over time, averaging 20.5% before 2013 Q3, at which point requests increased (Fig. S2). Of the 1,136 specimens received by the BCCDC PHL during the study period, only 271 (23.8%) had genotyping requested specifically to confirm or refute suspected transmission (Table 1) However, our subsequent universal genotyping analysis revealed an overall provincial genotypic clustering proportion of 38.0%, meaning that prior to universal genotyping, on-request genotyping captured fewer clusters.

**TABLE 1:** Reasons for 300^*a*^ genotype requests in BC from 2009 to 2013

Request reason	*n* (%)^*b*^
Transmission	
Specified patient comparison	41 (13.7)
Specified outbreak comparison	111 (37.0)
General database comparison	119 (39.7)
Relapse or reinfection	12 (4.0)
Specimen mix-up/cross-contamination	17 (5.7)

a Included are all patients who were subjects of genotyping requests (*n* = 296). Four patients were the subjects of multiple genotyping requests for different reasons; here, we count each request separately (*n* = 4).

b Percentages have been rounded and may not add up to 100%.

### Genotype requests reflected suspected community transmission and known risk factors.

Most requests (90.3%) were made during contact investigations to confirm or refute transmission, although few named specific individuals (Table 1). Instead, most requests asked for a comparison against a specific outbreak genotype or the general database. When a specific comparator was identified (*n* = 152 requests)—either a patient or a specific outbreak genotype—a match between the requested strain and comparator was observed in 83 instances (54.6%). When we examined all of the isolates requested to determine possible transmission, we found that 67.5% (183/271) matched at least one other isolate by MIRU-VNTR genotyping during the study period. Requests to differentiate relapse from reinfection (*n* = 12) and requests to investigate potential laboratory errors (*n* = 17) were less frequent.

We next compared the characteristics of patients for whom genotyping was requested to confirm or refute transmission (*n* = 269 after the exclusion of two individuals whose genotype was requested on more than one occasion to investigate transmission) versus all other patients in the study sample representing true positive TB diagnoses (Table 2). We found that proportionally more requests were made for individuals in the 35- to 54-year age group, males, those born in Canada, and persons with one or more risk factors (HIV, drug use, or alcohol misuse).

**TABLE 2:** Demographic characteristics of the study sample^*a*^ (*n* = 1,158) and comparison of patients whose isolates were requested for genotyping to confirm/refute transmission (*n* = 269) with all other samples (*n* = 889)

Characteristic	No. (%) with genotyping requested to confirm/refute transmission		*P* value^*b*^
	Yes	No	
Age, yr			
0–34	60 (23.6)	194 (76.4)	<0.001
35–54	111 (32.5)	231 (67.5)	
55–74	66 (21.5)	241 (78.5)	
75+	32 (12.5)	223 (87.5)	
Gender			
Female	101 (21.2)	376 (78.8)	0.188
Male	168 (24.7)	513 (75.3)	
Birthplace^*c*^			
Canada	158 (51.6)	148 (48.4)	<0.001
Outside Canada	105 (12.9)	709 (87.1)	
No. of risk factors^*d*^			
0	131 (16.6)	657 (83.4)	<0.001
≥1	70 (54.7)	58 (45.3)	

a We excluded false-positive TB diagnoses (*n* = 3) and counted each patient once by excluding the second record from reoccurrences (*n* = 14).

b Chi-square test.

c Data unavailable (*n* = 38).

d The risk factors are HIV, drug use, and alcohol misuse. Data unavailable for one or more risk factors, *n* = 242.

### Universal genotyping improves cluster identification.

Province-wide, retrospective universal genotyping (17) revealed how many clusters and how many clustered individuals were missed during the on-request period. From 2009 through 2013, 94 genotypic clusters were observed in BC, ranging in size from 2 to 53 cases (mean = 5) and involving a total of 432 individuals. On-request genotyping missed 54 (57.4%) of these clusters and 130 (30.1%) clustered individuals (Table 3).

**TABLE 3:** Characteristics of MIRU-VNTR genotyping clusters identified through universal genotyping categorized by the proportion of each cluster (none, partial, or all) requested for genotyping to confirm or refute potential transmission

Cluster requested proportion (%)	No. of clusters	No. (%) predominantly Canadian born	Cluster size range	Mean cluster size ±SD
None (0)	54	10 (18.5)	2–6	2.4 ± 0.8
Partial (1–99)	30	14 (46.7)	2–53	9.1 ± 10.7
All (100)	10	5 (50.0)	2–5	3.0 ± 1.2

**FIG 1:**
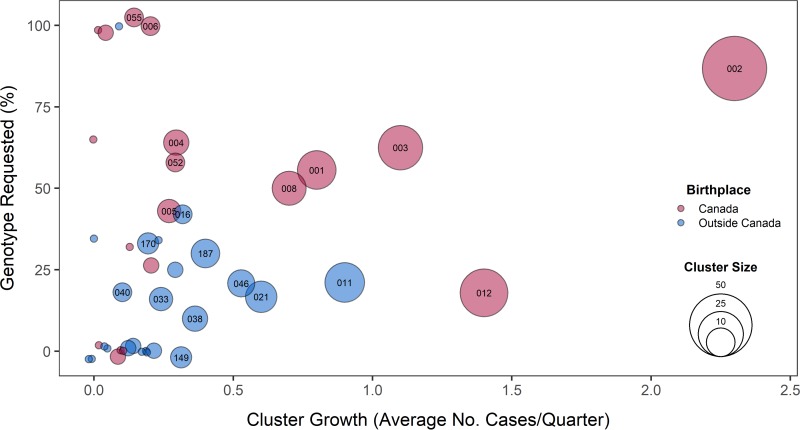
Bubble plot of the proportion of each cluster requested for genotyping to confirm or refute transmission, with the average cluster growth per quarter in BC from 2009 to 2013. Growing clusters had a minimum of three persons in the cluster over the study period. Bubbles are colored to indicate the predominant birthplace (≥50%) of the individuals in each cluster and sized to represent the total number of genotypically clustered cases. Cluster identifiers are indicated for clusters with five or more patients.

Ten clusters (10.6%), with an average of three patient isolates per cluster, were fully identified through on-request genotyping; 30 clusters (31.9%) were partially identified (Table 3; Fig. 1). These partial clusters tended to be larger (9.1 ± 10.7 persons/cluster) than those that were either missed or fully identified (≤6 persons/cluster). The mean proportion of requested cases within a partially identified cluster was 40.5%. Clusters described as predominantly Canadian born (*n* = 29) were more likely to be partially or fully requested (Table 3).

We used logistic regression analysis to examine the characteristics of those in genotypic clusters and found that individuals in the 35- to 54-year age group, males, those born in Canada, and persons with one or more risk factors (HIV, drug use, or alcohol misuse) were more likely to belong to a cluster than to have a unique genotype (Table 4). We observed that isolates that had a historical genotype request had greater odds of belonging to a genotypic cluster (aOR, 2.3; 95% CI, 1.5 to 3.3); this effect size increased (aOR, 3.3; 95% CI, 2.0 to 5.4) when we excluded the last two quarters of 2013 from the analysis (Table S1). We also examined risk factors in relation to genotype requests and clustering status and found that 258 (72.5%) of the 356 persons with clustered isolates had no risk factors identified (Table S2).

**TABLE 4:** Logistic regression analysis of the relationship between MIRU-VNTR genotypic clustering, as revealed by universal genotyping, and whether an isolate was originally requested for genotyping to confirm or refute transmission

Characteristic	Clustered^*a*^ vs unique OR (95% CI)	
	Unadjusted	Adjusted
Requested		
Yes	4.6 (3.3–6.5)	2.3 (1.5–3.3)
No	Reference	Reference
Age, yr		
0–34	Reference	Reference
35–54	1.7 (1.2–2.5)	1.5 (1.0–2.3)
55–74	0.9 (0.6–1.4)	1.0 (0.6–1.5)
75+	0.5 (0.3–0.8)	0.8 (0.5–1.3)
Gender		
Male	1.3 (1.0–1.7)	1.1 (0.8–1.5)
Female	Reference	Reference
Birthplace		
Canada	8.8 (6.2–12.3)	5.3 (3.5–7.8)
Outside Canada	Reference	Reference
No. of risk factors^*b*^		
0	Reference	Reference
≥1	6.6 (4.2–10.2)	1.8 (1.0–3.0)

a A cluster is ≥2 patients with the same genotype by 24-locus MIRU-VNTR genotyping.

b The risk factors are HIV, drug use, and alcohol misuse.

### Growing clusters were variably identified by on-request genotyping.

**FIG 2:**
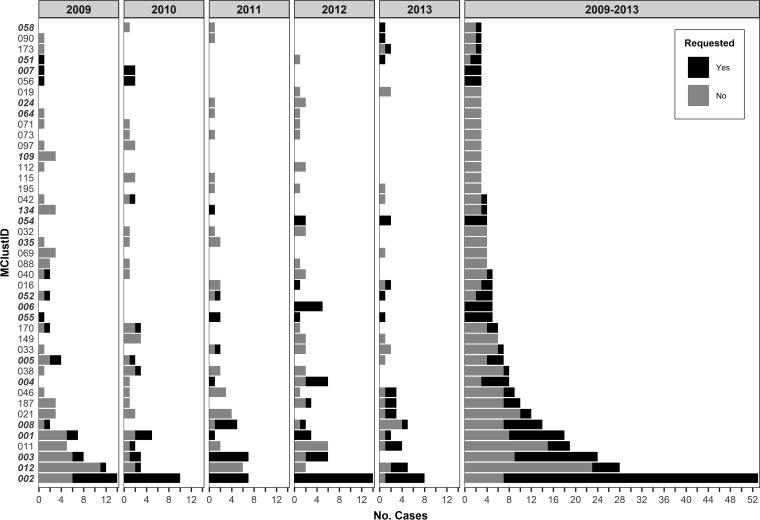
Annual cluster growth and overall cluster size for all clusters with three or more persons in BC from 2009 to 2013. Bars are colored by genotype requested (yes/no). Twenty-four-locus MIRU-VNTR genotyping cluster identifiers (MClustID) in bold italics represent clusters that are composed of predominantly Canadian-born persons.

To examine growing clusters, we pruned the data set to include only the 43 clusters with three or more persons and examined the cluster growth rate and the proportion of requested cases (Fig. 1 and 2). Although request rates varied, Canadian-born clusters with higher growth rates were larger and tended to have proportionally more isolates requested for genotyping (*P* = 0.003). MClust-002, a previously described TB outbreak in BC (22), was the largest cluster observed during the study period (*n* = 53) and had the highest average rate of growth (2.3 cases/quarter) and the largest number of clustered cases observed in a single quarter (*n* = 9). Within this cluster, an additional seven cases were identified through universal genotyping—six of these were early in the outbreak (2009 Q1). Two other recognized outbreaks, one previously described (3) (growth rate = 0.8 case/quarter) and the other spanning a more remote part of the province (1.1 cases/quarter), had partially requested isolates (44.4 and 37.5% of cases missed, respectively). MClust-012 involved an urban population with a high material deprivation index (Table S3). Here, only 5 of 28 individuals in the cluster had a genotyping request (Fig. 2; Table S3), 3 of which were late in the outbreak (2013), and the requests for a 2009 and a 2010 isolate asked for comparisons to outbreak strains other than MClust-012. Requests were less common among clusters involving largely foreign-born individuals, where the request rate in the three largest clusters (≥10 individuals) averaged 22.6% (Table S3).

## DISCUSSION

In low TB incidence settings, clinical laboratories considering universal genotyping must demonstrate that it offers substantial epidemiological insights beyond those from an on-request service. This study leveraged a unique situation, in which we compared 5 years of an on-request genotyping program to the information later gained from retrospective genotyping of all of the isolates during this period, to generate the evidence to justify ongoing universal genotyping.

During the on-request period, the existence of many genotypic clusters and the full extent of many other clusters were missed. MIRU-VNTR genotyping overestimates the clustering of related isolates, particularly for clusters involving non-Euro-American M. tuberculosis lineages (23). With 62% of BC's cases attributable to non-Euro-American lineages (17), some of our missed clusters are likely pseudoclusters and do not reflect true local transmission. However, clusters involving the Canadian born that do likely represent local transmission were also partially or fully missed by on-request genotyping. Whole-genome sequencing (WGS) of all of our clustered isolates is under way to provide a more accurate quantification of local transmission within BC, as well as strain-specific insights into drug resistance and transmissibility.

Genotyping requests were most often used to investigate suspected community transmission, particularly in individuals with known risk factors. MIRU-VNTR genotyping results confirmed many potential transmission events, but specific suspicions, in which an individual or outbreak strain comparator was noted in the request, were less frequently correct. This suggests that clinicians understood the risk factors for transmission but that the underlying epidemiological networks were not as clear. Universal genotyping provides a bias-free method to identify connections between cases and reveal possible transmissions between individuals who do not fit traditional risk profiles.

In a secondary analysis, restricting the data to include only dates prior to the spike in requests (2013 Q3 and Q4) increased the odds of belonging to a genotypic cluster in relation to request status. These results indicate a possible shift in reasoning behind genotype requests in 2013. Clinicians were likely recognizing that genotyping provides a deeper understanding of the molecular epidemiology of TB and were thus issuing genotyping requests not only to address a specific hypothesis about transmission but also to understand the overall transmission dynamics of TB in BC.

Prospective universal genotyping will enable earlier detection of clusters and allow prompt intervention (14). However, this can only occur if those capable of acting on the information have timely access to it. Universal genotyping requires an efficient and effective means of communicating genotyping results, such as the online tools developed in other jurisdictions (7, 24). While implementation of a universal genotyping program incurs additional costs, we believe that the incremental expenditure associated with additional genotyping and the cost of implementing a new reporting system are minimal on the scale of a provincial public health budget. This is especially true when considered in the context of TB infections prevented, as the average cost of treating a person with active TB in Canada is $47,000 (25), and when universal genotyping refutes suspected transmission and large-scale contact tracing and case finding are avoided, especially in complex settings such as homeless shelters (14). Tangible benefits are also realized when specimen cross-contamination events are revealed by universal genotyping and a patient can be taken off unnecessary therapy (26, 27).

While our data make a strong case for implementing universal genotyping in a low-incidence setting, it is impossible to know with certainty how many new infections would have been prevented if universal genotyping had been in place since 2009; thus, we are unable to assess the true public health impact of this intervention. However, universal genotyping of M. tuberculosis in New York City revealed new transmission sites and contributed to the rapid diagnosis and treatment of both active cases and infected contacts (14). It is also difficult to assess the future potential of universal genotyping in well-resourced settings, where WGS is supplanting MIRU-VNTR genotyping as the method of choice for inferring transmission. Until WGS of all isolates is routinely performed, MIRU-VNTR genotyping and other molecular methods provide valuable insight into a region's TB epidemiology and permit comparison of patterns across jurisdictional boundaries. If countries like Canada are to achieve the ambitious elimination targets set by the World Health Organization, every available tool in our arsenal must be used to accelerate progress toward making TB an infection of the past.

## Supplementary Material

Supplemental material
