# Supplementary material for: Universal Genotyping for Tuberculosis Prevention Programs: a 5-Year Comparison with On-Request Genotyping
Source: J Clin Microbiol. 2018 Apr 25;56(5):e01778-17. doi: 10.1128/JCM.01778-17 (PMC5925716; doi:10.1128/JCM.01778-17)
Supplement: Supplemental material [file supp_56_5_e01778-17__index.html]

Universal Genotyping for Tuberculosis Prevention Programs: a 5-Year Comparison with On-Request Genotyping — Supplemental material 

# Universal Genotyping for Tuberculosis Prevention Programs: a 5-Year Comparison with On-Request Genotyping

## Supplemental material

- Supplemental file 1 -

  Fig. S1 (Venn diagram representing genotype request statuses of study sample) and S2 (Percentages of isolates for which genotyping was requested) and Tables S1 (Logistic regression analysis for relationship between MIRU-VNTR genotypic clustering and genotype request status), S2 (Relationship between genotype request status, risk factors, and genotypic clustering), and S3 (Characteristics of 24-locus MIRU–VNTR clusters comprising ≥5 individuals)

  PDF, 414K
