# Supplementary material for: Universal Genotyping for Tuberculosis Prevention Programs: a 5-Year Comparison with On-Request Genotyping
Source: J Clin Microbiol. 2018 Apr 25;56(5):e01778-17. doi: 10.1128/JCM.01778-17 (PMC5925716; doi:10.1128/JCM.01778-17)
Supplement: Supplemental material [file JCM.01778-17_zjm999095946s1.pdf]

SUPPLEMENTAL MATERIAL

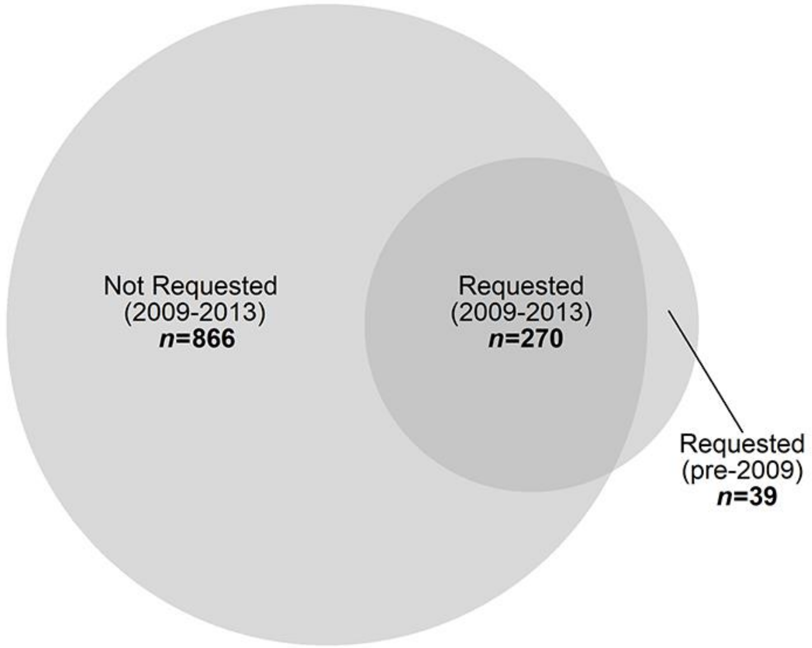

FIG S1 Venn diagram representing the request status of the study sample (*n*=1,175) for isolates with genotyping requested (all reasons) from 2009 through 2013, which included all genotyped isolates from specimens received at the British Columbia Centre for Disease Control Public Health Laboratory from 2009 through 2013, and those requested for genotyping with a specimen received date prior to 2009.

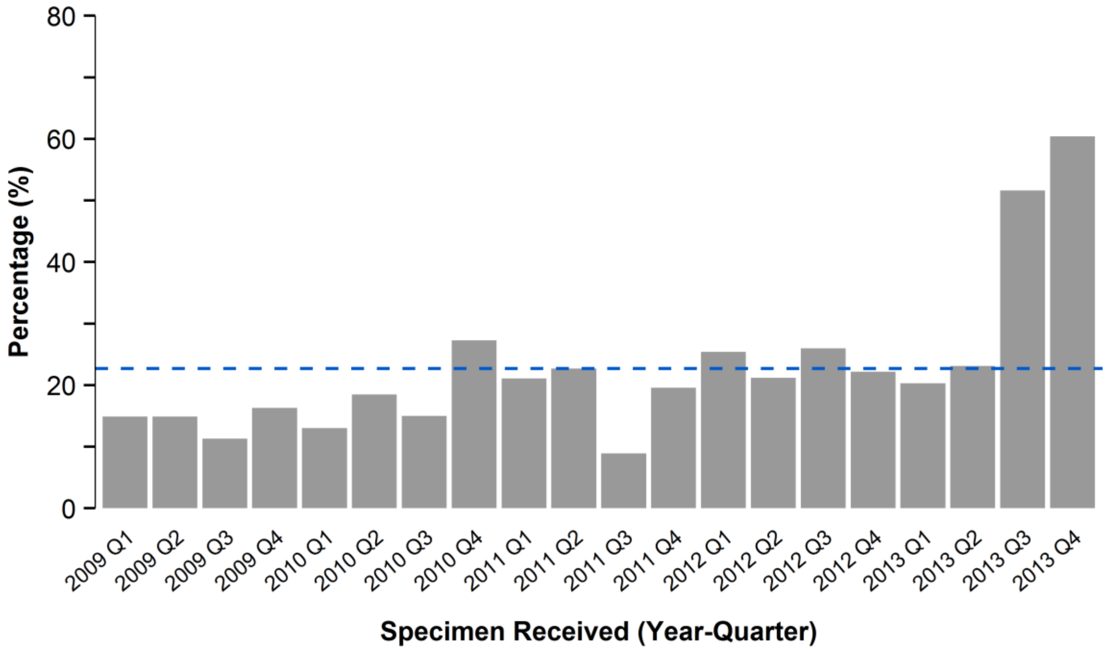

FIG S2 Percentage of isolates requested for genotyping (all reasons) displayed by year and quarter specimens were received at the British Columbia Centre for Disease Control Public Health Laboratory from 2009 through 2013. Dashed line represents the mean percentage of genotyped isolates requested per quarter.

TABLE S1 Logistic regression analysis for the relationship between MIRU-VNTR genotypic clustering and genotyping requested (2009–2013Q2) to confirm or refute transmission (*n*=813), British Columbia.

	Clustered[a] vs. Unique	
Characteristic	Unadjusted OR (95%CI)	Adjusted OR (95%CI)
Requested		
Yes	8.5 (5.5–13.1)	3.3 (2.0–5.4)
No	Reference	Reference
Age, years		
0-34	Reference	Reference
35-54	1.8 (1.3–2.7)	1.6 (1.0–2.5)
55–74	1.0 (0.6–1.4)	1.0 (0.6–1.6)
75+	0.5 (0.3–0.8)	0.8 (0.4–1.3)
Gender		
Male	1.2 (0.9–1.6)	1.0 (0.7–1.4)
Female	Reference	Reference
Birthplace		
Canada	9.1 (6.3–13.0)	4.9 (3.2–7.3)
Outside Canada	Reference	Reference
Risk Factors[b]		
None	Reference	Reference
≥1	6.8 (4.3–10.8)	1.8 (1.0–3.1)

OR = odds ratio; CI = confidence interval.
[a]*Cluster: ≥ 2 patients that share an identical genotype (24-locus MIRU-VNTR).*
[b]*Risk Factors = HIV positive, drug use, or alcohol misuse.*

TABLE S2 Relationship between genotype request status, risk factors[a] and genotypic clustering (Yes/No), British Columbia, 2009–2013.

		Clustered (No.)	
Characteristic	No. Isolates (%)	Yes	No
Requested			
No Risk Factors	131 (65.2)	73	58
≥1 Risk Factors	70 (34.8)	62	8
Not Requested			
No Risk Factors	657 (91.9)	185	472
≥1 Risk Factors	58 (8.1)	36	22

[a]*Risk Factors = HIV, drug use, or alcohol misuse; data unavailable for 1 or more risk factor (n=242).*

TABLE S3 Characteristics of 24-locus MIRU–VNTR clusters comprised of ≥5 individuals, displayed as clusters that were predominantly Canadian- or foreign-born, British Columbia, 2009–2013.

Cluster ID	Cluster Size (% Requested)	Predominant Birthplace[a] (%)	Median Age (IQR) years	Gender M:F	Predominant Community Type (%)	Risk Factors[b] (%)	Median Deprivation[c] Quintile
Canadian–born							
MClust-002	53 (86.8)	Canada (86.0)	51 (44–58)	12.2	Metro (71.7)	39.6	4.0
MClust-012	28 (17.9)	Canada (89.3)	46 (41–51)	1.8	Metro (89.3)	35.7	4.0
MClust-003	24 (62.5)	Canada (95.7)	44 (28–52)	2.4	Urban/Rural (41.7/41.7)	45.8	4.0
MClust-001	18 (55.6)	Canada (100.0)	46 (37–52)	0.8	Rural (50.0)	44.4	4.0
MClust-008	14 (50.0)	Canada (85.7)	51 (37–63)	1.3	Metro (85.7)	42.9	3.0
MClust-004	8 (62.5)	Canada (100.0)	47 (40–57)	1.0	Urban (75.0)	37.5	4.0
MClust-005	7 (42.9)	Canada (85.7)	57 (46–60)	2.5	Metro (85.7)	14.3	2.0
MClust-006	5 (100.0)	Canada (100.0)	25 (23–37)	0.7	Rural (80.0)	0.0	3.0
MClust-052	5 (60.0)	Canada (100.0)	53 (43–61)	NA[d]	Metro/Urban (40.0/40.0)	100.0	5.0
MClust-055	5 (100.0)	Canada (100.0)	42 (34–48)	0.7	Urban (80.0)	20.0	3.0
Foreign-born							
MClust-011	19 (21.1)	Philippines (94.4)	42 (33–53)	2.8	Metro (68.4)	0.0	4.0
MClust-021	12 (16.7)	Philippines (100.0)	44 (29–51)	0.7	Metro (83.3)	0.0	3.5
MClust-187	10 (30.0)	*Mixed*[e] (88.8)	80 (55–88)	0.7	Metro (100.0)	0.0	3.0
MClust-046	9 (22.2)	India (77.8)	70 (56–75)	1.2	Metro (88.9)	0.0	2.0
MClust-038	8 (12.5)	China/Hong Kong (100.0)	79 (72–81)	3.0	Metro (100.0)	0.0	3.5
MClust-033	7 (14.3)	*Mixed*[e] (85.8)	68 (42–76)	0.8	Metro (100.0)	0.0	3.0
MClust-149	6 (0.0)	India (83.3)	50 (34–76)	0.5	Metro/Urban (50.0/50.0)	0.0	2.5
MClust-170	6 (33.3)	China (83.3)	68 (55–75)	1.0	Metro (100.0)	0.0	3.0
MClust-016	5 (40.0)	Philippines (100.0)	39 (32–41)	0.7	Metro/Urban (40.0/40.0)	0.0	3.0
MClust-040	5 (20.0)	*Mixed*[e] (80.0)	75 (35–86)	0.7	Metro (100.0)	0.0	3.0

IQR: interquartile range.

[a]*Information for birthplace was unknown for 6 patients; percentage represents those with complete data.*

[b]*One or more risk factors for transmission (HIV, drug use, or alcohol misuse); data unavailable (n=50); percentage represents those with complete data.*

[c]*Canadian Marginalization Index (20), material deprivation (quintile 1: least deprived, quintile 5: most deprived); data unavailable (n=23).*

[d]*All individuals were male.*

[e]*Various Asian countries.*
